# A novel single-catheter access device for endoscopic ultrasound-guided hepaticogastrostomy

**DOI:** 10.1055/a-2971-5161

**Published:** 2026-10-12

**Authors:** Christina S Gainey, Judy A Trieu, Kelly E Hathorn, Todd H. Baron

**Affiliations:** 1 Department of Gastroenterology and Hepatology 2331 The University of North Carolina at Chapel HillChapel HillNorth CarolinaUnited States

Endoscopic ultrasound–guided hepaticogastrostomy (EUS-HGS) is well established for malignant biliary obstruction when endoscopic retrograde cholangiopancreatography (ERCP) fails or is not feasible. 1​ Large cohorts have validated its efficacy and durability, 2​ 3​ ​4​ but the standard technique relies on multiple exchanges: puncture, guidewire placement, tract dilation or cystotome cautery, and stenting. Loss of tract access is a critical risk. We demonstrate EUS-HGS using a single-catheter access device (Rivos; Boston Scientific, Marlborough, Massachusetts, USA) that integrates a 19-gauge trocar tip, an access cannula with an echogenic segment, and an electrocautery tip, shown ex vivo and in use ( Video 1 ).

**Video 1** EUS-guided hepaticogastrostomy for malignant biliary obstruction using a single-catheter access device: trocar puncture, cholangiography, guidewire coiling, electrocautery tract creation, and covered metal stent placement with coaxial double-pigtail stent.


A 64-year-old man with metastatic pancreatic adenocarcinoma presented with malignant biliary obstruction. Magnetic resonance imaging/magnetic resonance cholangiopancreatography showed a 10.2-cm pancreatic mass with an abrupt stricture of the common hepatic duct and severe intrahepatic biliary dilatation ( Fig. 1 ). Given the bulky mass and dilated ducts, primary EUS-guided drainage was chosen over ERCP to reduce post-ERCP pancreatitis risk; published data support this approach. 5​

**Fig. 1:**
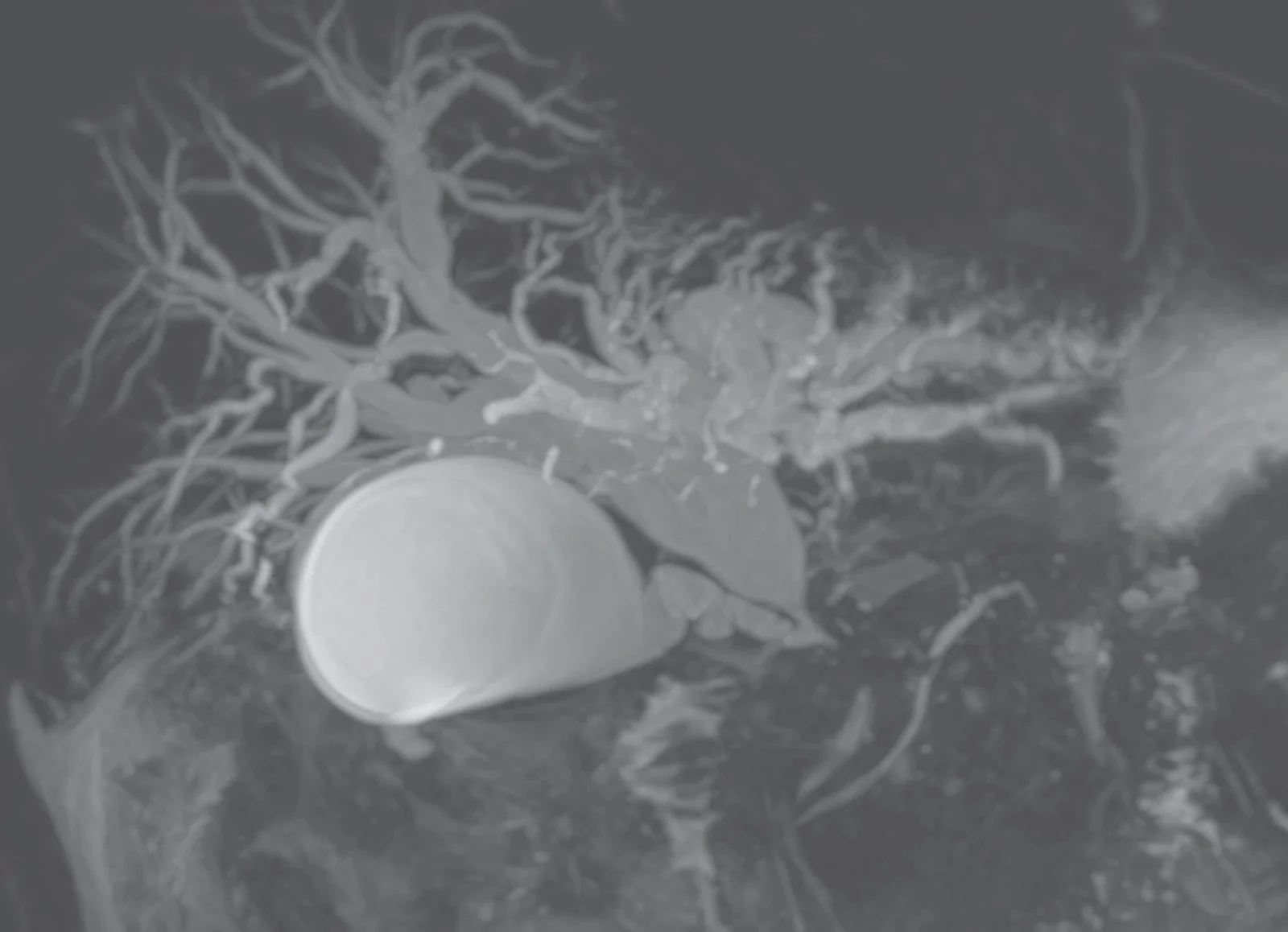
Magnetic resonance cholangiopancreatography showing the abrupt stricture of the common hepatic duct with severe upstream dilatation of the intrahepatic bile ducts.

Linear EUS showed dilated left intrahepatic ducts of up to 10 mm ( Fig. 2 )and color Doppler confirmed an avascular window for puncture ( Fig. 3 ). With the catheter locked at the working channel, the trocar punctured the duct. Cholangiography verified intraductal position and proximal obstruction.

**Fig. 2:**
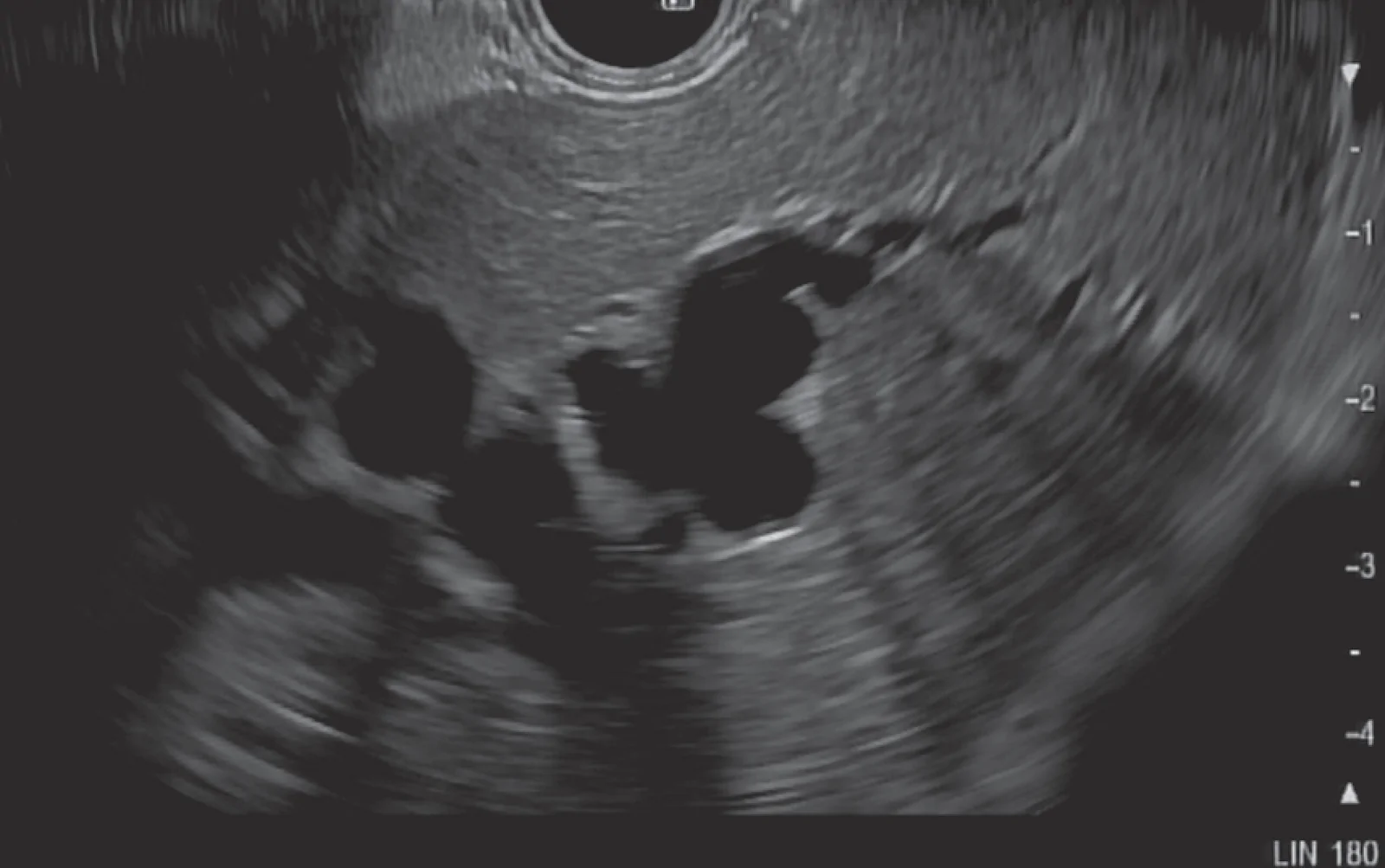
An endoscopic ultrasound view from the proximal stomach showing the dilated left intrahepatic bile ducts.

**Fig. 3:**
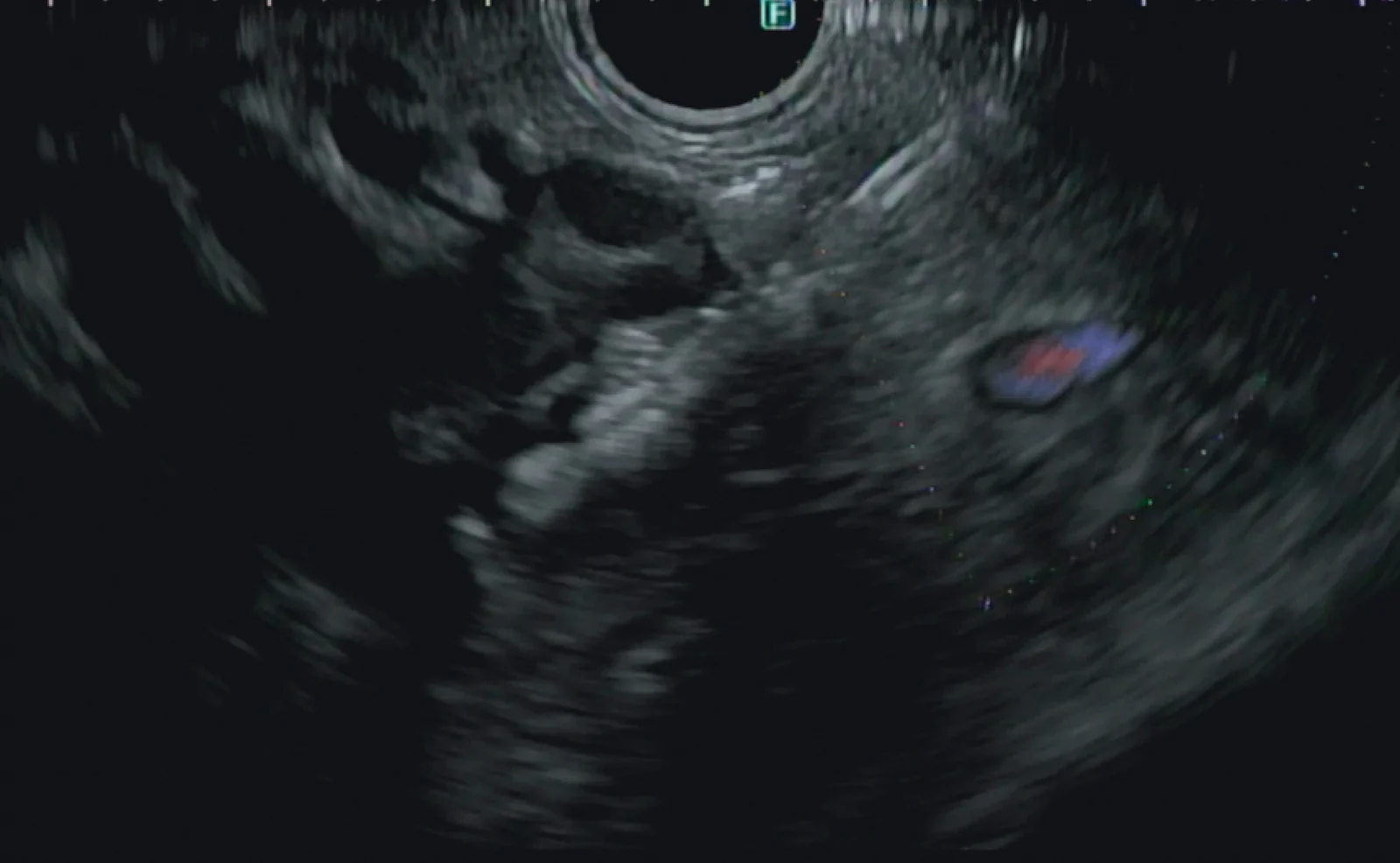
Color Doppler interrogation of the common wall between the stomach and the dilated left intrahepatic duct, confirming an avascular window.

Following needle withdrawal, a 0.025-inch guidewire was coiled within the biliary tree through the flexible polymer access cannula, so the wire never contacted a metal needle bevel, an usual cause of shearing. The integrated electrocautery tip created the tract, eliminating device exchange. A 10-mm×6-cm fully covered metal stent (Viabil, WL Gore, Flagstaff, AZ, USA) was deployed with a coaxial 7 Fr double-pigtail stent ( Figs. 4 5 ). Bile drainage was immediate without adverse events, and the patient was discharged the same day.

**Fig. 4:**
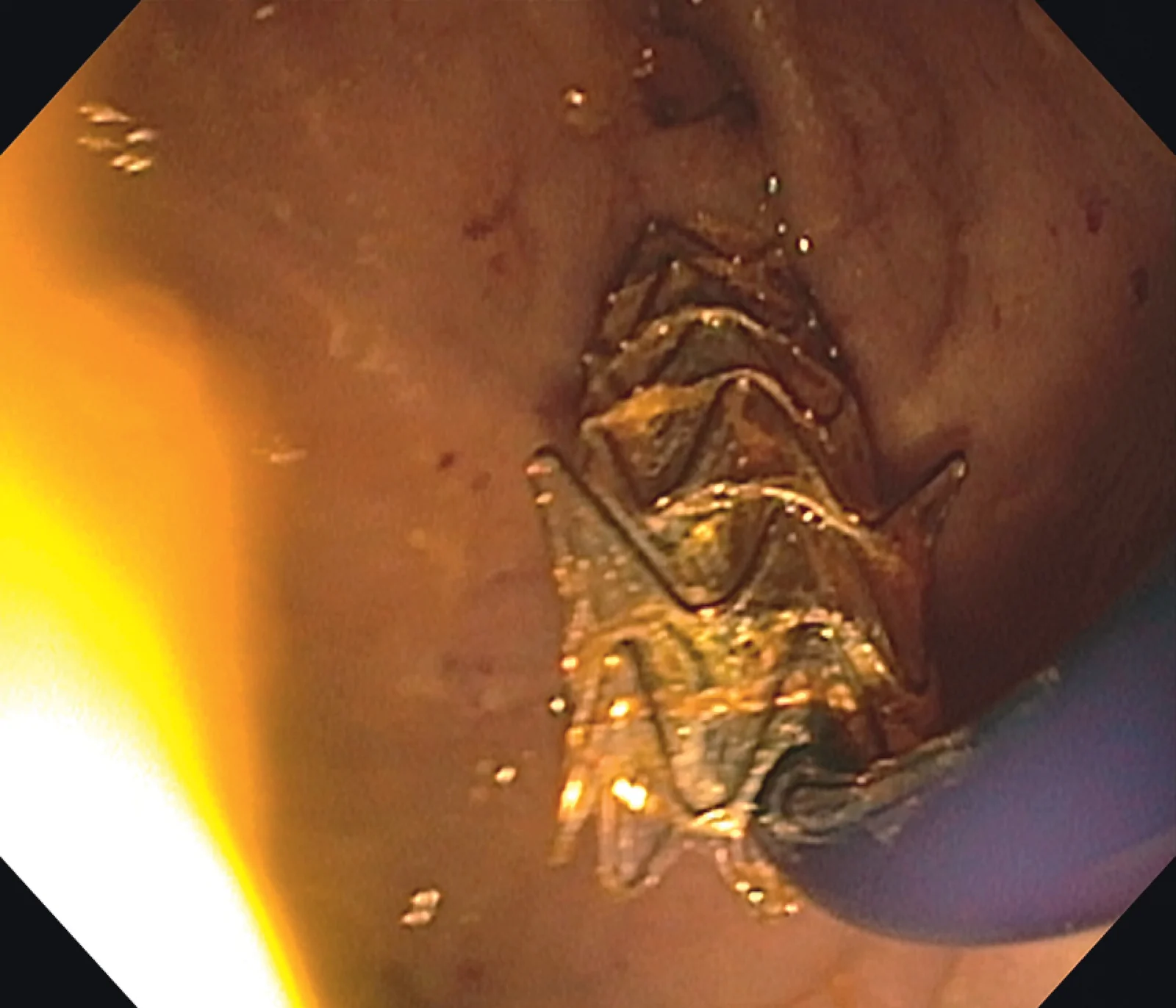
An endoscopic view of the gastric end of the fully covered metal stent traversing the hepaticogastrostomy.

**Fig. 5:**
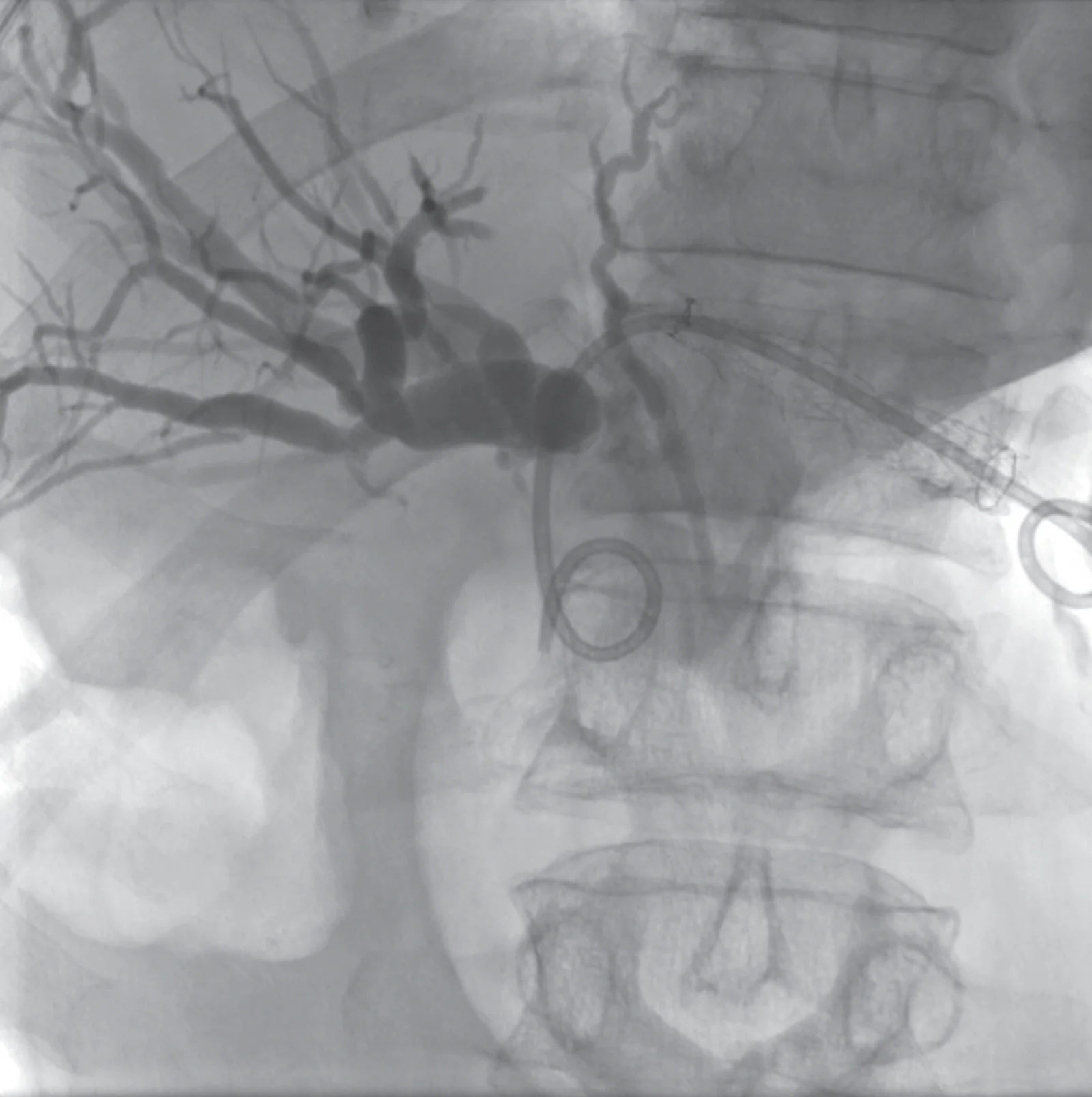
A final fluoroscopic image: a fully covered metal stent across the hepaticogastrostomy with a coaxial double-pigtail stent.

By consolidating puncture, guidewire access, and tract creation into a single instrument, this device may reduce the risk of losing transmural access.

Endoscopy_UCTN_Code_TTT_1AS_2AH

## Declaration of GenAI Use

The authors used Claude (Anthropic) for language editing of the manuscript text. Generative artificial intelligence was not used to generate, alter or enhance any clinical data, endoscopic images, fluoroscopic images or radiological images, and was not used to generate scientific contents or interpretation. The authors reviewed and verified all texts and images and take full responsibility for the integrity and accuracy of the work.
